# Development of a workflow for identification of nuclear genotyping markers for *Cyclospora cayetanensis*

**DOI:** 10.1051/parasite/2020022

**Published:** 2020-04-10

**Authors:** Katelyn A. Houghton, Alexandre Lomsadze, Subin Park, Fernanda S. Nascimento, Joel Barratt, Michael J. Arrowood, Erik VanRoey, Eldin Talundzic, Mark Borodovsky, Yvonne Qvarnstrom

**Affiliations:** 1 Parasitic Diseases Branch, Division of Parasitic Diseases and Malaria, Center for Global Health, Centers for Disease Control and Prevention Atlanta GA 30329 USA; 2 Malaria Branch, Division of Parasitic Diseases and Malaria, Center for Global Health, Centers for Disease Control and Prevention Atlanta GA 30329 USA; 3 Waterborne Disease Prevention Branch, Division of Foodborne, Waterborne, and Environmental Diseases, National Center for Emerging and Zoonotic Infectious Diseases, Centers for Disease Control and Prevention Atlanta GA 30329 USA; 4 Wallace H. Coulter Department of Biomedical Engineering, Georgia Institute of Technology Atlanta GA 30332 USA

**Keywords:** Cyclosporiasis, *Cyclospora cayetanensis*, Genotyping

## Abstract

*Cyclospora cayetanensis* is an intestinal parasite responsible for the diarrheal illness, cyclosporiasis. Molecular genotyping, using targeted amplicon sequencing, provides a complementary tool for outbreak investigations, especially when epidemiological data are insufficient for linking cases and identifying clusters. The goal of this study was to identify candidate genotyping markers using a novel workflow for detection of segregating single nucleotide polymorphisms (SNPs) in *C. cayetanensis* genomes. Four whole *C. cayetanensis* genomes were compared using this workflow and four candidate markers were selected for evaluation of their genotyping utility by PCR and Sanger sequencing. These four markers covered 13 SNPs and resolved parasites from 57 stool specimens, differentiating *C. cayetanensis* into 19 new unique genotypes.

## Abstract (fr)

*Cyclospora cayetanensis* est un parasite intestinal responsable de la cyclosporose, maladie diarrhéique. Le génotypage moléculaire, utilisant le séquençage ciblé des amplicons, fournit un outil complémentaire pour les enquêtes sur les épidémies, en particulier lorsque les données épidémiologiques sont insuffisantes pour relier les cas et identifier les grappes. Le but de cette étude était d’identifier des marqueurs candidats de génotypage à l’aide d’un nouveau flux de travail pour la détection des polymorphismes d’un seul nucléotide (SNP) différentiateurs dans les génomes de *C. cayetanensis*. Quatre génomes entiers de *C. cayetanensis* ont été comparés à l’aide de ce flux de travail et quatre marqueurs candidats ont été sélectionnés pour l’évaluation de leur utilité de génotypage par PCR et séquençage Sanger. Ces quatre marqueurs couvraient 13 SNP et ont résolu les parasites provenant de 57 spécimens de selles, différenciant *C. cayetanensis* en 19 nouveaux génotypes uniques.

## Introduction

The coccidian parasite *Cyclospora cayetanensis*, identified as a cause of food-borne diarrheal illness in the early 1990s, is routinely linked to sporadic cases and annual, seasonal outbreaks of cyclosporiasis [27]. In the United States, there were over 2000 domestically acquired cases reported in 2018 alone and over half of them were not linked to a contaminated food vehicle [3]. Epidemiologic investigations are the primary method for identifying clusters of cases in food-borne illness; however, they are the only tool available for cyclosporiasis since there is no validated molecular typing tool. Molecular typing tools are routinely used to support outbreak investigations for other intestinal illnesses [12, 17]. Challenges in developing a typing tool are multifactorial for *C. cayetanensis*. Currently, there is no method to propagate this parasite for routine laboratory study [8]. To study *C. cayetanensis* in the laboratory, the parasite must be obtained from infected patients’ stool and purified [24], which is a laborious process. This often leads to only picogram levels of DNA for library preparation [20], which is not sufficient input for whole genome sequencing (WGS). The isolation of genomic DNA from *C. cayetanensis,* once the parasite is obtained, has also been difficult. The structure of the thick oocyst wall has required specialized extraction methods to obtain DNA fragment lengths sufficient for WGS, while a notably large genome (~44 MB) [23] has made obtaining whole genome sequences difficult and unfeasible as a routine genotyping approach.

Recent advances in whole genome sequencing of *C. cayetanensis* [4, 18, 23, 28] facilitated the initial identification of potential genotyping markers. Three studies described the use of a multilocus sequence typing (MLST) method based on microsatellites [10, 13, 16], an approach successfully applied to other parasites [29]. The first of these three studies observed different sequence types based on geography, but this method was not evaluated for its usefulness in epidemiologic case linkage [10]. The second and third MLST studies noted poor resolution due to a high proportion of unreadable sequences [13, 16]. An alternative method to the MLST approach identified a hypervariable region in the mitochondrial genome as a genotyping marker, due to its high diversity among parasites [9] and high copy number [28]. However, Guo and colleagues only reported the success in geographical segregation with no discussion of resolving regional outbreak clusters [11]. Nascimento and colleagues resolved nearly 84% of samples epidemiologically linked to outbreak clusters using the proposed mitochondrial marker [19]. A third approach targeting three genomic regions of high entropy, possessing several single nucleotide polymorphisms (SNPs) and an algorithm to predict sample relatedness, resolved four of eight epidemiologically linked outbreak clusters [2].

These genotyping methods show promise; however, none have yet been adopted for routine use due to their limited ability to fully resolve the diverse and complex nature of cyclosporiasis outbreak clusters. Additional markers may be required to further improve these methods and capture the genetic variability between *C. cayetanensis* outbreak samples. Thus, the goal of this study was to develop a new workflow to identify additional SNPs in the nuclear genome of *C. cayetanensis,* and subsequently, provide additional markers for genotyping.

## Materials and methods

### Gene identification pipeline development

Raw Illumina sequencing reads from eight whole genomes known as CDC:HCNY16:01 (Accession: GCA_001305735.1), CDC:TX69:14 (Accession: GCA_002019455.1), CDC:HCRI001:97 (Accession: GCA_002019905.1), CDC:HCGM01:97 (Accession: GCA_002019465.1), CDC:HCDC004_96 (Accession: GCA_003945175.1), CHN_HEN01 (Accession: GCA_000769155.2), CDC:HCNP016_97 (Accession: GCA_003945145.1) and CDC:HCJK001:14 (Accession: GCA_002019475.1) were used for the workflow development and marker generation. Quality of the sequencing reads was evaluated by FASTQC v0.11.7 [1] and bases with Phred scores less than four were removed. Paired reads overlapping (by > 30 nt) were merged by AdaptorRemoval v 2.2.2 [26]. The human genome assembly GRCh38.p12 was used to filter out reads mapping to the human genome. To identify possible contaminants, all the contigs shorter than 10K nt were aligned by BLASTN against the NCBI NT database. The trimmed paired reads were aligned by STAR v 2.5.4b (in “no-intron” mode) to GenBank reference genomes of the identified contaminant species. All reads that mapped to contaminant genomes were filtered out. Remaining reads were *de novo* assembled into draft *C. cayetanensis* genome assembly using SPAdes v3.11.1 [21]. Additionally, all *de novo* assembled contigs were aligned by BLASTN to mitochondria and apicoplast sequences of *C. cayetanensis*; only contigs of nuclear DNA origin were included in the final genome assembly.

Annotation of protein coding genes in nuclear DNA was performed by the GeneMark-EP+ gene finding tool, https://www.biorxiv.org/content/10.1101/2019.12.31.891218v2. GeneMark-EP+ utilizes cross-species protein splice alignments generated by ProSplign [15] to a genome of interest as external information (homologous protein footprints) in both model parameter estimation (training) and gene prediction steps. The reference set of proteins for the GeneMark-EP+ algorithm was the set of Apicomplexa proteins from the EggNOG v4.5 database [14]. Protein footprints (hints) were generated from spliced alignments of reference proteins to genomic DNA. Next, the full run of GeneMark-EP+ generated gene predictions. Functional annotation of the genes predicted in the eight *C. cayetanensis* genomes was made by the Blast2Go algorithm [6].

A Mauve algorithm [7] was used to align assembled *C. cayetanensis* genomes and to identify syntenic regions and positions of SNPs. To increase the reliability of SNP calling, base calling quality was calculated for each base in the assembly. All the reads were aligned by the STAR algorithm to the assembled genomic sequences. Each base was characterized by read coverage and frequency of dominant base call.

To further narrow down the marker search space, only four genomes with highest read coverage and, arguably, with higher quality of assembly were selected (CDC:HCNY16:01, CDC:TX69:14, CDC:HCRI001:97, and CDC:HCGM01:97, see Supplementary materials). Additional filtering criteria required i) single copy protein-coding genes; ii) genes with significant similarity (at least 70% identity) to homologous Apicomplexa proteins as detected by BLAST search; iii) syntenic genomic regions present in all analyzed strains; iv) SNPs with 99% dominant base and minimum read coverage 20; and v) regions with at least three SNPs within 400 nucleotide span (regardless of exon borders). The resulting list was searched for genes that had SNPs in a single isolate, e.g. CDC:HCNY16:01, with no SNP present in the other three isolates CDC:TX69:14, CDC:HCRI001:97 and CDC:HCGM01:97. The identified candidate genes, with at least 70% of their protein products to known Apicomplexa proteins, were then ranked by the number of observed SNPs. The highest ranked candidate in each of the four genomes was selected for further analysis. Primers for these four regions were designed using Primer3 [25] with the goal of capturing as many SNPs as possible within a “PCR friendly” length (Table 1).

**Table 1:** Characteristics of the four primer sets used to amplify the marker regions.

Target name	Primer name	Primer sequence (5′–3′)	*T*_m_	Hairpin T_m_	Target	Amplicon length (bp)	Haplotypes	SNPs
CDS-1	GT1-F	CTCCTTGCTGCTCAGAACGA	60	none	ATP synthase	175	2	7
	GT1-R	CAAGAGAGGAGCAGTGGCAA	60	44.6				
CDS-2	GT2-F	TGCAAACTACTAAGGGCGCA	60	none	U3 small nucleolar RNA-associated protein 11 ((LOC34621252)	246	2	1
	GT2N-R	CGCCTTCTCTTGAGCCTTGA	60	none				
CDS-3	GT3-F	AATCGAATCGGTGCAGTGCTTA	60.7	none	Uncharacterized (LOC34620832)	220	3	2
	GT3N-R	GACTGAACGTGTGAGAGGGG	59.3	none				
CDS-4	GT4-F	GTAGATGGGTCCTTGAAGGCT	59.2	none	ATP-dependent RNA helicase rrp3 (LOC34619020)	179	2	3
	GT4N-F	CAGACGCCTAAGGAACCGAA	60	37.6				

### Molecular methods

The four chosen markers were evaluated using 93 *C. cayetanensis*-positive stool specimens collected from 2013, 2014, 2015, and 2017. Due to the low volume and availability of some specimens, not all genes were tested on all 93 specimens. The samples had been sent to the Centers for Disease Control and Prevention (CDC) by US State health departments for research purposes. They were received unpreserved, suspended in non-nutritive media (e.g., Cary-Blair transport medium) or preserved in alcohol-based fixatives (e.g., TOTAL-FIX, Medical Chemical Corporation, Torrance, CA), and used in accordance with the Human Research Protection Office in the Center for Global Health, Centers for Disease Control and Prevention, “Use of coded specimens for *Cyclospora* genomics research” (2014-107). The presence of oocysts was confirmed by epifluorescence microscopy.

Samples were washed free of preservative through one to three rounds of centrifugation at 2500 ×*g* for three minutes with phosphate-buffered saline (pH 7.2) and diluted to form a thick slurry. Nucleic acid was extracted using the UNEX-based method [22] and subjected to conventional PCR for amplification of the four marker gene fragments (Table 1). The fragments were amplified in a 25 μL PCR reaction using NEBNext Q5 Hot Start HiFi PCR Master Mix (New England Biolabs, Ipswich, MA), 400 nM each of the forward and reverse primers, and 1 μL of the DNA template. The cycling conditions included an initialization step at 98 °C for 2 min, followed by 35 cycles of 98 °C for 15 s denaturing, 67 °C for 15 s annealing, and 65 °C for 15 s extension. The final extension was set to 65 °C for 5 min. PCR products were visualized on a 1.5% agarose gel stained with ethidium bromide (Applied Biosystems, Foster City, CA).

The PCR products were purified using Monarch^®^ PCR and DNA Cleanup Kit (New England Biolabs, Ipswich, MA) and sequenced on an ABI PRISM^®^ 3130xl Genetic Analyzer (Applied Biosystems, Foster City, CA) in both directions using the PCR primers and the BigDye Terminator V3.1 chemistry (Applied Biosystems, Foster City, CA). The DyeEx 2.0 Spin Kit (Qiagen, Hilden, Germany) was used to remove unincorporated dyes before sequencing (Qiagen, Hilden, Germany).

DNA sequences were visualized and analyzed within Geneious v 11.1.2 (Auckland, New Zealand). Identification of underlying haplotypes for each marker gene was performed as described previously [2] to create consensus haplotype references for each marker. Forward and reverse ABI sequence files for each sample were trimmed using a Phred quality score of 30 and an error probability limit of 0.05, then aligned to the haplotype reference file for each marker. Heterozygous bases were identified in the alignment with the Geneious Heterozygote Plug-in v1.5.1, with a 25% peak similarity threshold. Bases identified through the Heterozygote Plug-in were then manually inspected for double peak verification. Bray-Curtis dissimilarity values were calculated and plotted using a hierarchical agglomerative clustering method [5] to visualize the relationship between samples and their haplotypes.

## Results

### Gene identification pipeline

The set of *C. cayetanensis* specific gene prediction parameters was determined by training of the GeneMark-EP+ gene prediction algorithm on the CDC:HCNY16:01 isolate genome. The protein mapping pipeline, a part of GeneMark-EP+, was executed for all the assemblies. Final gene prediction was completed using the *C. cayetanensis* specific parameters together with isolate specific protein hints to predict genes. More than 80,000 SNP positions were detected using the Mauve genome alignment algorithm. Application of the filtering criteria produced a set of 485 genes candidates. These 485 genes were narrowed down to regions that had SNPs in a single isolate with no SNP present in any of the other three isolates. In each genome, at least five such genes were identified. After the candidate genes were ranked by the number of observed SNPs and similarity score to the known Apicomplexa proteins, the highest ranked candidate in each of the four genomes was selected for further analysis. This search resulted in four marker regions that uniquely identified genomes of four isolates (see Supplementary materials for full marker sequence and SNP locations).

### Marker gene evaluation

The four chosen markers (labeled CDS-1, CDS-2, CDS-3, and CDS-4) were evaluated by testing *C. cayetanensis*-positive stool specimens collected during 2013, 2014, 2015, and 2017. Of the 93 specimens available for testing, 84 were tested with CDS-1, 83 with CDS-2, 73 with CDS-3, and 78 with CDS-4. Successful amplification and sequencing for all four targets combined was accomplished in 57 of the stool specimens, with 114 from CDS-1, 104 from CDS-2, 86 from CDS-3, and 109 from CDS-4. Individual marker sequencing success rates were 61% for CDS-1, 77% for CDS-2, 75% for CDS-3, and 74% for CDS-4 (data for marker success calculated from ongoing laboratory studies). Sequence information and SNP locations for each haplotype can be found in Supplementary materials. Representative nucleotide sequences for each marker genes’ haplotypes were deposited into GenBank. Accession numbers are as follows: MN367319, MN367320, MN367321, MN367322, MN367323, MN367324, MN367325, MN367326, and MN367327.

**Figure 1:**
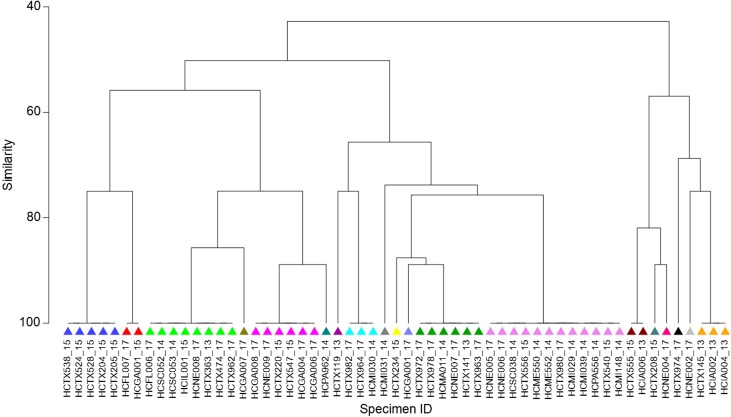
Cluster dendrogram, using Bray-Curtis values, to visualize diverse potential of the four markers described here. This figure demonstrates the amount of variability captured through the combination of these four markers and that they were able to resolve 57 specimens into 19 distinct genotypes.

A presence-absence table was generated of all four marker haplotypes present in each sample, with a 1 if the haplotype was present and a 0 if absent. If a sample had a true double peak, indicating a mixed haplotype infection, the sample had a 1 recorded for both haplotypes in that marker. To aid in visualizing the relationship between specimens, Bray-Curtis dissimilarity values were calculated from the presence-absence table of haplotypes and plotted (Fig. 1) using a hierarchical agglomerative clustering method [5].

## Discussion

The four markers were successfully amplified in 57 *C. cayetanensis* positive stool specimens collected in the United States from 2013 to 2017. At least two unique haplotypes were detected for each marker, with three haplotypes detected at the CDS-3 locus (Table 1). When combining all observed haplotypes for each marker, 19 unique genotypes were identified (Fig. 1). Ten of the 19 genotypes were represented in only one specimen, while the remaining genotypes were represented by two or more specimens. The most common genotype included 12 specimens that were collected from 2014 to 2017 from different geographical regions of the US (NE, SC, TX, ME, MI, and PA). The second most common genotype was comprised of eight specimens from 2013 to 2017 and again across different geographical regions of the US (FL, SC, IL, NE, TX). All genotypes, apart from one that was seen in all five specimens from Texas in 2015, were identified across a range of years and geographic locations in the US. Out of the 57 specimens evaluated in this study, five possessed double peaks at one or more SNP sites in the Sanger chromatograms for some markers (Fig. 1). Double peaks identified by Sanger sequencing indicate either sequence heterozygosity or a mixed infection; however, Sanger sequencing alone is insufficient to resolve the underlying haplotypes in some circumstances and a targeted NGS approach is needed to further resolve these genotypes.

This study utilized a newly described workflow for identification of SNP-rich nuclear markers that could supplement currently available *C. cayetanensis* genotyping tools. While only four markers were evaluated here, the workflow identified 481 additional markers, and further candidates may be identified by including additional genomes in the workflow. The four markers evaluated here were identified through the comparison of four draft *C. cayetanensis* genomes that represented those of the highest quality available at the time. These markers were able to discriminate between the four genomes utilized and resolved 57 specimens into 19 unique genotypes. Once further genomes of sufficient coverage and read depth for accurate SNP calling are available, this approach may be used to identify further candidate markers.

Individually, published genotyping methods for *C. cayetanensis* provide limited resolution of epidemiologic outbreak clusters, as on their own these panels may capture an insufficient amount of diversity [2, 13, 19]. A typing method that includes more markers, possibly including some derived from the mitochondrion and apicoplast, alongside those evaluated here, may provide the additional resolution required for a functional tool that can aid in outbreak investigations. We therefore propose that this small panel of markers may be used in conjunction with previously published panels to provide increased resolution of *C. cayetanensis* genotypes in the future.

## Conflict of interest

The authors have no conflicts of interest to disclose.

## Funding

This study was supported by the CDC’s Advanced Molecular Detection and Response to Infectious Disease Outbreaks (AMD) Initiative.

## Disclaimer

The findings and conclusions in this report are those of the author(s) and do not necessarily represent the official position of the Centers for Disease Control and Prevention.

## Supplementary materials

Supplementary materials are available as “Supplemental.xlsx” at https://www.parasite-journal.org/10.1051/parasite/2020022/olm. *Tab (Haplotypes)*: This table lists the DNA sequences for all haplotypes of all four markers, with primer binding sites and SNP sites highlighted.
*Tab (Sequence Data Preparation)*: Genome information, including size, number of contigs, and number of protein coding genes present in each of the four genomes used for the workflow.
*Tab (CDS-1)*: Sequence of whole marker gene found for the GM genome and its associated SNP coordinates.
*Tab (CDS-2)*: Sequence of whole marker gene found for the NY genome and its associated SNP coordinates.
*Tab (CDS-3)*: Sequence of whole marker gene found for the TX genome and its associated SNP coordinates.
*Tab (CDS-4)*: Sequence of whole marker gene found for the RI genome and its associated SNP coordinates.
*Tab (Haplotype presence-absence)*: Table including the presence or absence of each haplotype for all 57 specimens sequenced.
